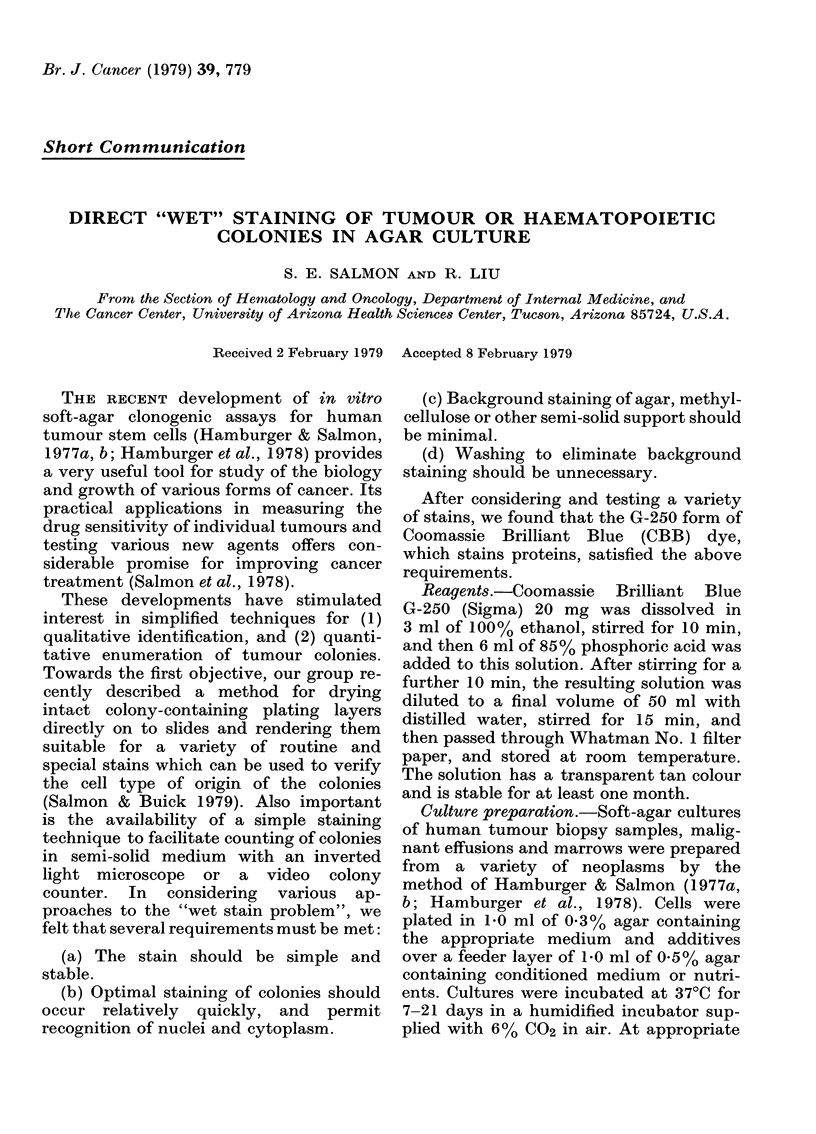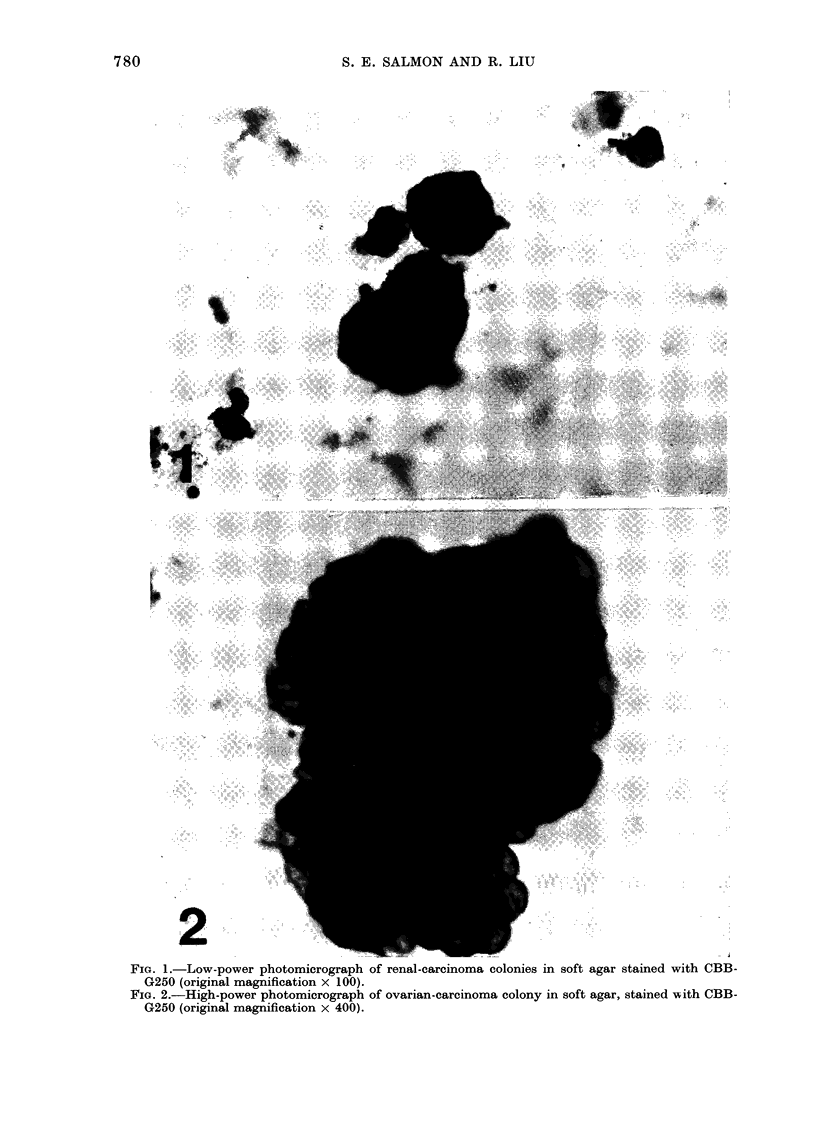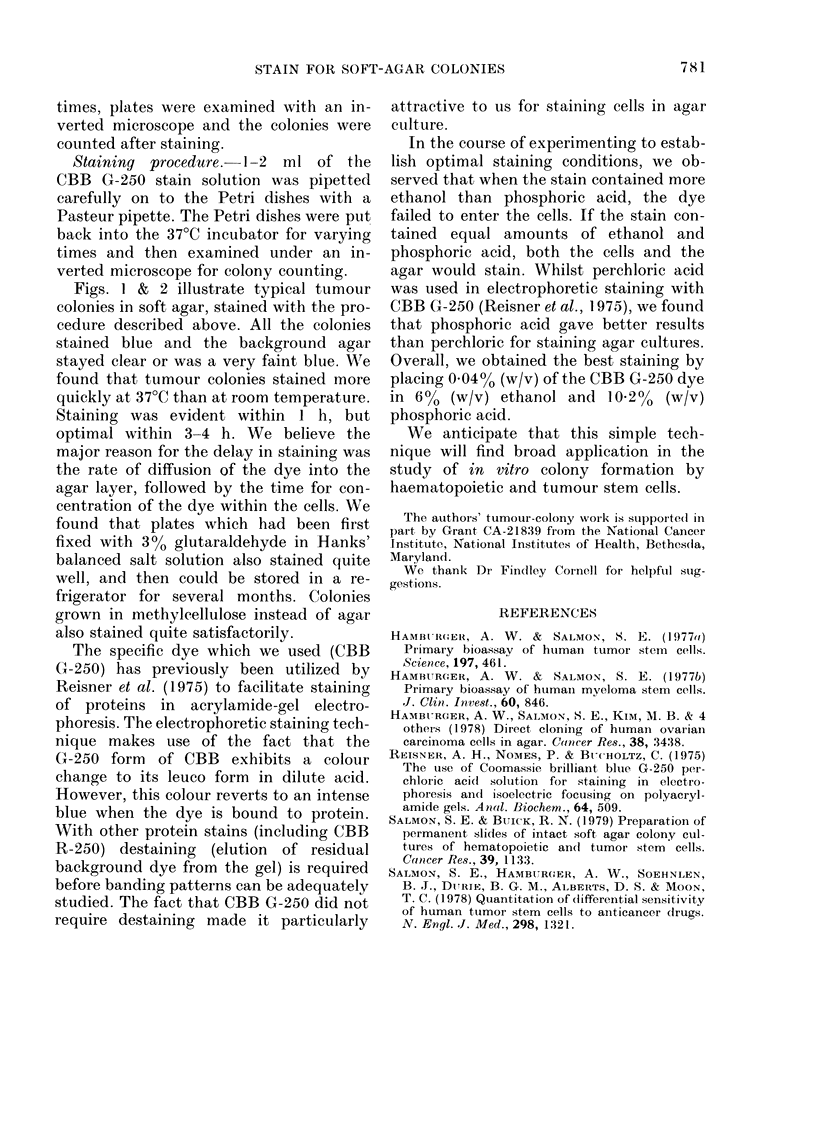# Direct "wet" staining of tumour or haematopoietic colonies in agar culture.

**DOI:** 10.1038/bjc.1979.132

**Published:** 1979-06

**Authors:** S. E. Salmon, R. Liu

## Abstract

**Images:**


*Br. J. Cancer* (1979) **39**, 779

***Short Communication***

### DIRECT "WET" STAINING OF TUMOUR OR HAEMATOPOIETIC COLONIES IN AGAR CULTURE

S. E. SALMON AND R. LIU

*From the Section of Hematology and Oncology, Department of Internal Medicine, and The Cancer Center, University of Arizona Health Sciences Center, Tucson, Arizona* 85724, *U.S.A.*

Received 2 February 1979 Accepted 8 February 1979

THE RECENT development of *in vitro* soft-agar clonogenic assays for human tumour stem cells (Hamburger & Salmon, 1977*a*, *b*; Hamburger *et al*., 1978) provides a very useful tool for study of the biology and growth of various forms of cancer. Its practical applications in measuring the drug sensitivity of individual tumours and testing various new agents offers considerable promise for improving cancer treatment (Salmon *et al*., 1978).

These developments have stimulated interest in simplified techniques for (1) qualitative identification, and (2) quantitative enumeration of tumour colonies. Towards the first objective, our group recently described a method for drying intact colony-containing plating layers directly on to slides and rendering them suitable for a variety of routine and special stains which can be used to verify the cell type of origin of the colonies (Salmon & Buick 1979). Also important is the availability of a simple staining technique to facilitate counting of colonies in semi-solid medium with an inverted light microscope or a video colony counter. In considering various approaches to the "wet stain problem", we felt that several requirements must be met:

(a) The stain should be simple and stable.

(b) Optimal staining of colonies should occur relatively quickly, and permit recognition of nuclei and cytoplasm.

(c) Background staining of agar, methylcellulose or other semi-solid support should be minimal.

(d) Washing to eliminate background staining should be unnecessary.

After considering and testing a variety of stains, we found that the G-250 form of Coomassie Brilliant Blue (CBB) dye, which stains proteins, satisfied the above requirements.

*Reagents*.—Coomassie Brilliant Blue G-250 (Sigma) 20 mg was dissolved in 3 ml of 100% ethanol, stirred for 10 min, and then 6 ml of 85% phosphoric acid was added to this solution. After stirring for a further 10 min, the resulting solution was diluted to a final volume of 50 ml with distilled water, stirred for 15 min, and then passed through Whatman No. 1 filter paper, and stored at room temperature. The solution has a transparent tan colour and is stable for at least one month.

*Culture preparation*.—Soft-agar cultures of human tumour biopsy samples, malignant effusions and marrows were prepared from a variety of neoplasms by the method of Hamburger & Salmon (1977*a*, *b*; Hamburger *et al*., 1978). Cells were plated in 1·0 ml of 0·3% agar containing the appropriate medium and additives over a feeder layer of 1·0 ml of 0·5% agar containing conditioned medium or nutrients. Cultures were incubated at 37°C for 7–21 days in a humidified incubator supplied with 6% $CO_2$ in air. At appropriate

FIG. 1.—Low-power photomicrograph of renal-carcinoma colonies in soft agar stained with CBB-G250 (original magnification × 100).

FIG. 2.—High-power photomicrograph of ovarian-carcinoma colony in soft agar, stained with CBB-G250 (original magnification × 400).

times, plates were examined with an inverted microscope and the colonies were counted after staining.

*Staining procedure.*—1–2 ml of the CBB G-250 stain solution was pipetted carefully on to the Petri dishes with a Pasteur pipette. The Petri dishes were put back into the 37°C incubator for varying times and then examined under an inverted microscope for colony counting.

Figs. 1 & 2 illustrate typical tumour colonies in soft agar, stained with the procedure described above. All the colonies stained blue and the background agar stayed clear or was a very faint blue. We found that tumour colonies stained more quickly at 37°C than at room temperature. Staining was evident within 1 h, but optimal within 3–4 h. We believe the major reason for the delay in staining was the rate of diffusion of the dye into the agar layer, followed by the time for concentration of the dye within the cells. We found that plates which had been first fixed with 3% glutaraldehyde in Hanks' balanced salt solution also stained quite well, and then could be stored in a refrigerator for several months. Colonies grown in methylcellulose instead of agar also stained quite satisfactorily.

The specific dye which we used (CBB G-250) has previously been utilized by Reisner *et al.* (1975) to facilitate staining of proteins in acrylamide-gel electrophoresis. The electrophoretic staining technique makes use of the fact that the G-250 form of CBB exhibits a colour change to its leuco form in dilute acid. However, this colour reverts to an intense blue when the dye is bound to protein. With other protein stains (including CBB R-250) destaining (elution of residual background dye from the gel) is required before banding patterns can be adequately studied. The fact that CBB G-250 did not require destaining made it particularly attractive to us for staining cells in agar culture.

In the course of experimenting to establish optimal staining conditions, we observed that when the stain contained more ethanol than phosphoric acid, the dye failed to enter the cells. If the stain contained equal amounts of ethanol and phosphoric acid, both the cells and the agar would stain. Whilst perchloric acid was used in electrophoretic staining with CBB G-250 (Reisner *et al.*, 1975), we found that phosphoric acid gave better results than perchloric for staining agar cultures. Overall, we obtained the best staining by placing 0·04% (w/v) of the CBB G-250 dye in 6% (w/v) ethanol and 10·2% (w/v) phosphoric acid.

We anticipate that this simple technique will find broad application in the study of *in vitro* colony formation by haematopoietic and tumour stem cells.

The authors' tumour-colony work is supported in part by Grant CA-21839 from the National Cancer Institute, National Institutes of Health, Bethesda, Maryland.

We thank Dr Findley Cornell for helpful suggestions.

### REFERENCES

HAMBURGER, A. W. & SALMON, S. E. (1977*a*) Primary bioassay of human tumor stem cells. *Science*, **197**, 461.

HAMBURGER, A. W. & SALMON, S. E. (1977*b*) Primary bioassay of human myeloma stem cells. *J. Clin. Invest.*, **60**, 846.

HAMBURGER, A. W., SALMON, S. E., KIM, M. B. & 4 others (1978) Direct cloning of human ovarian carcinoma cells in agar. *Cancer Res.*, **38**, 3438.

REISNER, A. H., NOMES, P. & BUCHOLTZ, C. (1975) The use of Coomassie brilliant blue G-250 perchloric acid solution for staining in electrophoresis and isoelectric focusing on polyacrylamide gels. *Anal. Biochem.*, **64**, 509.

SALMON, S. E. & BUICK, R. N. (1979) Preparation of permanent slides of intact soft agar colony cultures of hematopoietic and tumor stem cells. *Cancer Res.*, **39**, 1133.

SALMON, S. E., HAMBURGER, A. W., SOEHNLEN, B. J., DURIE, B. G. M., ALBERTS, D. S. & MOON, T. C. (1978) Quantitation of differential sensitivity of human tumor stem cells to anticancer drugs. *N. Engl. J. Med.*, **298**, 1321.